# Herbivore-specific induction of indirect and direct defensive responses in leaves and roots

**DOI:** 10.1093/aobpla/plz003

**Published:** 2019-02-01

**Authors:** Li Xiao, Juli Carrillo, Evan Siemann, Jianqing Ding

**Affiliations:** 1 Key Laboratory of Aquatic Plant and Watershed Ecology, Wuhan Botanical Garden, Chinese Academy of Sciences, Wuhan, China; 2 University of Chinese Academy of Sciences, Beijing, China; 3 Faculty of Land and Food Systems, Centre for Sustainable Food Systems, Biodiversity Research Centre, University of British Columbia, Vancouver, British Columbia, Canada; 4 Biosciences Department, Rice University, Houston, TX, USA; 5 School of Life Sciences, Henan University, Kaifeng, Henan, China

**Keywords:** Above- and below-ground interactions, extrafloral nectar, herbivory, secondary chemicals, tallow tree, trade-offs

## Abstract

Herbivory can induce both general and specific responses in plants that modify direct and indirect defence against subsequent herbivory. The type of induction (local versus systemic induction, single versus multiple defence induction) likely depends both on herbivore identity and relationships among different responses. We examined the effects of two above-ground chewing herbivores (caterpillar, weevil) and one sucking herbivore (aphid) on indirect defence responses in leaves and direct defence responses in both leaves and roots of tallow tree, *Triadica sebifera.* We also included foliar applications of methyl jasmonate (MeJA) and salicylic acid (SA). We found that chewing herbivores and MeJA increased above-ground defence chemicals but SA only increased below-ground total flavonoids. Herbivory or MeJA increased above-ground indirect defence response (extrafloral nectar) but SA decreased it. Principal component analysis showed there was a trade-off between increasing total root phenolics and tannins (MeJA, chewing) versus latex and total root flavonoids (aphid, SA). For individual flavonoids, there was evidence for systemic induction (quercetin), trade-offs between compounds (quercetin versus kaempferitrin) and trade-offs between above-ground versus below-ground production (isoquercetin). Our results suggest that direct and indirect defence responses in leaves and roots depend on herbivore host range and specificity along with feeding mode. We detected relationships among some defence response types, while others were independent. Including multiple types of insects to examine defence inductions in leaves and roots may better elucidate the complexity and specificity of defence responses of plants.

## Teaser

Few studies have investigated to what degree herbivory can induce both highly specific and broad chemical changes in plants across different defence classes. This study examined the effects of multiple herbivores and two plant signalling hormones on indirect defence and direct defence responses in leaves and roots of *Triadica sebifera*. We provide evidence that the different herbivores can induce highly specific plant responses; moreover, the simultaneous and relative induction of different defence types depends on the herbivore identity and plant part (roots versus shoots).

## Introduction

Herbivory-induced defensive responses in plants can be direct (e.g. secondary chemicals suppressing herbivory) or indirect (e.g. extrafloral nectar [EFN] attracting ants) (Howe and Jander 2008; Hagenbucher *et al.* 2013; Kaplan *et al.* 2016; Aljbory and Chen 2018). These different types of anti-herbivore responses may vary temporally (time after damage) and spatially (e.g. roots versus leaves). Since plant resources are limited, theory suggests that plants face allocation trade-offs in generating induced defensive responses, and that the expression of response traits will depend on allocational, evolutionary and ecological costs (Heil 2002; Bekaert *et al.* 2012; van Velzen and Etienne 2015; Züst *et al.* 2015). These relationships are complex, including synergistic interactions among defensive responses (Agrawal and Fishbein 2006), antagonistic trade-offs (Koricheva *et al.* 2004; Kempel *et al.* 2011; Koricheva and Romero 2012; Moles *et al.* 2013; Haak *et al.* 2014) or some combination of positive and negative interactions (Agrawal 2011; Agrawal *et al.* 2014).

Potentially complicating the detection of relationships among defensive response types is the observation that plant responses can be species-specific, affected by both herbivore identity and feeding type (Rodriguez-Saona *et al.* 2010; Carrillo *et al.* 2012; Gutbrodt *et al.* 2012; Wang *et al.* 2013; Gu *et al.* 2014; de Oliveira *et al.* 2016), and can be locally or systemically induced (van de Ven *et al.* 2000; Zarate *et al.* 2007). In nature, plants are often attacked by an array of herbivorous insects that vary in feeding mode (chewing versus sucking), location (above- versus below-ground) or timing, which can shape induced responses in plants (Carrillo *et al.* 2012; Wang *et al.* 2014). Plants are thought to generally respond to chewing insects through induction of jasmonic acid (JA)-mediated pathways while sucking insects are considered to generally induce salicylic acid (SA)-mediated responses (Reymond and Farmer 1998; Walling 2000). However, even similarly feeding insects that cause equivalent amounts of damage can induce different or equivalent responses, depending on the type of response trait measured (Carrillo *et al.* 2014). Thus, including multiple types of insects and simultaneously measuring multiple plant defensive responses to these insects may indicate the relative specificity of responses in general and better reveal relationships among different defence traits (Rasmann *et al.* 2009). Such studies, however, are rare (Huang *et al.* 2014).

Knowledge of local and systemic induction may be particularly important for better understanding the relationships among multiple defensive responses, such as those induced by feeding above-ground versus below-ground, or by different herbivore types. Feeding in one location may induce systemic changes in the response levels in other plant parts (Huang *et al.* 2013, 2015; Wondafrash *et al.* 2013), and induced-indirect responses, such as plant volatiles or EFN production, can share induction pathways with direct defence responses (Thaler *et al.* 2002; Cortés *et al.* 2016; Sanches *et al.* 2017). Given that the synthesis of defensive chemicals is often determined by associated signal transmission between leaves and roots (Bezemer and van Dam 2005; Erb *et al.* 2008; Kaplan *et al.* 2008a, b) and plant induction response can be herbivore species-specific (Schmidt *et al.* 2005; Gutbrodt *et al.* 2012; Huang *et al.* 2014), we expect that above- and below-ground induction of defensive responses will depend on both herbivory type and identity. However, the relative specificity of herbivore induction of multiple defence responses and across multiple herbivore types is unknown. Even for individual defence classes, for example EFN, it is unclear when defence induction will be broadly versus narrowly induced. Root herbivory (Huang *et al.* 2015) and artificial root damage (Carrillo and Siemann 2016) can increase leaf EFN production, even in the absence of an above-ground herbivory cue. In contrast to this broad induction pattern, previous studies have also shown herbivore-specific induction of EFN, with chewing herbivores inducing stronger defence responses than sucking insects (Carrillo *et al.* 2012).

Herbivore induction of plant direct and indirect defensive responses may also be time-dependent, complicating measurement of induction across different herbivore types. For example, in *Plantago lanceolata*, the concentration of direct defensive chemicals increased over the time period from 1 and 8 days after herbivore damage (Wang *et al.* 2015), while herbivore-damaged cabbage plants induced indirect defences within 1 h after infestation (Scascighini *et al.* 2005). Several studies showed a time difference in attractiveness to parasitoids with induction by herbivory versus methyl jasmonate (MeJA) application (Thaler 1999; Mattiacci *et al.* 2001; Bruinsma *et al.* 2009), suggesting that the temporal scale of plant defence responses varies with induction type. To fully understand the specificity of defensive responses to different herbivores, measurements across different time scales are likely necessary but seldom done for multiple herbivores or multiple defensive responses.

Here we use *Triadica sebifera* and multiple herbivorous insects that vary in feeding mode as a system to examine relationships among different defensive responses induced above- and below-ground. Previous studies show that *T. sebifera* possesses multiple anti-herbivore responses such as secondary chemicals (Wang *et al.* 2012a; Huang *et al.* 2013, 2014), latex (Gu *et al.* 2014) and EFN (Carrillo *et al.* 2012; Wang *et al.* 2013) and these responses vary with plant populations (Carrillo *et al.* 2012; Wang *et al.* 2012a; Gu *et al.* 2014). In this study, we conducted a common garden experiment to evaluate plant responses to above-ground feeding by two chewing herbivores (caterpillar, weevil) and a sucking herbivore (aphid). We also applied MeJA and SA to induce plant responses dependent on those signalling pathways. Specifically, we ask: (i) How do direct (total phenolics, total tannins, flavonoids, latex) and indirect defensive responses (EFN) vary among herbivores? (ii) Which defensive responses to herbivores are independent, positively correlated or negatively correlated? (iii) How do these induction patterns vary through time?

## Materials and Methods

### Study organisms

*Triadica sebifera* (Euphorbiaceae) is a perennial tree native to Asia and cultivated as an oil and ornamental plant in many areas of southern China (Zhang and Lin 1994). It is an aggressively invasive plant in the USA (Pile *et al.* 2017).

*Heterapoderopsis bicallosicollis* (Coleoptera: Attelabidae) is a weevil with a narrow host range which can cause severe damage to *T. sebifera* plants in its native range (Wang *et al.* 2009) It is multivoltine with adults overwintering in soil litter and beginning oviposition in the spring (Wang *et al.* 2009). Adult females form sealed leaf rolls (nidi) where oviposition, larval development and pupation occur. After eclosion, adults emerge from the nidi and feed on new vegetative growth.

*Gadirtha inexacta* (Lepidoptera: Noctuidae) is a potential biological control agent against *T. sebifera* (Wang *et al.* 2012b), as it is a multivoltine specialist caterpillar which feeds only on leaves of *T. sebifera* plants. In its last three instars, the larvae can cause serious leaf damage and can result in complete defoliation in field conditions.

*Toxoptera odinae* (Hemiptera: Aphididae) is a generalist aphid that feeds on new growth and tends to cluster and attach to soft, green stems. Infestations of aphids develop quickly as they are highly mobile and travel rapidly from one plant to another. *Toxoptera odinae* is frequently observed feeding on *T. sebifera* plants in its native range (Zheng *et al.* 2005; Zhang *et al.* 2015).

We collected *H. bicallosicollis* and *T. odinae* adults from *T. sebifera* plants in Wuhan, China, for use in the following experiments. We collected larvae of *G. inexacta* from a field in Wuhan, reared them on local *T. sebifera* plants and used their offspring for experiments.

### Seeds and seedlings

We conducted all experiments at Wuhan Botanical Garden in 2015. We hand-collected seeds from Wuhan *T. sebifera* populations in November 2014. We planted seeds in a greenhouse on 9 April 2015. We individually transplanted similar-sized (6–8 fully expanded leaves) seedlings into pots containing top soil collected from a field without *T. sebifera* plants and arranged them in a greenhouse on 16 June 2015. In order to protect against naturally recruiting herbivores, we enclosed each plant within a nylon mesh cage (100 cm height; 27 cm diameter).

### Induction experiment

To compare the specific responses of plants to different types of induction at different times after induction, we used three different herbivore species (*H. bicallosicollis*, *G. inexacta* and *T. odinae*) to damage the seedlings and two exogenous defence response-related plant hormones (MeJA and SA) to elicit an induced defence response. For the induction treatments: (i) we placed two *H. bicallosicollis* weevil adults on plants and removed them when ~25 % leaf area had been consumed (1–2 days of feeding); (ii) we placed two *G. inexacta* caterpillar larvae on plants and removed them when ~25 % leaf area had been consumed (estimated visually and occurring after 1–2 days of feeding); (iii) we inoculated plants with 50 *T. odinae* aphid adults and removed them with a soft brush after 2 days; (iv) we sprayed plants with a solution of 0.1 mmol L^−1^ MeJA (#39270; Sigma-Aldrich, St. Louis, MO, USA) in deionized water with ethanol 2.5 % (v:v); (v) we sprayed plants with a solution of 1 mmol L^−1^ SA (#84210; Sigma-Aldrich) in deionized water with ethanol 2.5 % (v:v); (vi) we sprayed one group of control plants (for MeJA and SA treatments) with the carrier solution of deionized water with ethanol 2.5 % (v:v); and (vii) we did not damage or spray another group of control plants (for herbivore treatments). We sprayed MeJA, SA and ethanol control plants evenly over the foliage with a hand-held sprayer until run-off occurred (~1 mL per leaf, ~25 mL per plant). To avoid cross-contamination, we applied spray treatments in separate chambers, and then placed these sprayed plants back with the herbivore treatments and controls.

We started the induction treatments on 3 September 2015, when each plant had ~25 fully expanded leaves. In a pilot study, we sprayed plants with different concentrations of MeJA (0.01, 0.1 and 1 mmol L^−1^) and SA (0.1, 1 and 10 mmol L^−1^), and found induction peaks for total phenolics and total flavonoid responses occurring on day 3 (D3, with D1 being the day of induction), day 6 (D6) and day 10 (D10) with 0.1 mmol L^−1^ MeJA and 1 mmol L^−1^ SA. Thus, we timed our harvest for these days post induction in the main experiment, although for some defences and damage stimuli we had no prior information about induction timing. In total, there were 105 plants (7 induction treatments × 3 harvest times × 5 replicates).

### EFN and latex measurements

To calculate the relative odds of a leaf producing EFN, we recorded the number of total leaves and the number of these leaves with nectaries producing EFN. We collected all EFN of each plant with a 0.3-mm-diameter glass micro-capillary tube, and measured the length of the EFN in the glass capillary with a Vernier caliper to calculate the volume of EFN.

For latex measurement, we cut off three fully expanded, undamaged leaves of each plant from the leaf base, and immediately after clipping each leaf, collected the latex exudate with a 1-cm sterile filter paper disc (no. 1; Whatman International, Maidstone, Kent, UK). We weighed each disc to the nearest microgram before or after latex collection, and calculated the weight difference as latex exudation.

### Chemical analyses

We harvested leaves and roots of each plant, flash froze them with liquid nitrogen and stored them at −20 °C for subsequent chemical analysis. We dried leaf and root samples of each plant in a vacuum freeze dryer (LGJ-10 Vacuum Freeze Dryer, Ningbo Xinyi Ultrasound Equipment Co. Ltd, Ningbo, Zhejiang, China) for 2 days and then ground them in a ball mill. We extracted each 100 mg sample with a methanol–0.4 % phosphoric acid in water solution (48:52, v:v) and filtered the solutions through a 0.22-µm membrane to remove insoluble material.

To estimate total tannin content, we used a modified radial diffusion assay (Hagerman 1987). We placed the filtered extracts (60 µL) of each sample in a 5-mm-diameter well in 1 % (wt v^−1^) agarose gel plate with 0.1 % (wt v^−1^) bovine serum albumin, and measured the precipitated protein area of each well after incubation for 3 days at 30 °C. We used tannic acid (Sigma-Aldrich) as a standard. For total phenolic content estimation, we used the modified Prussian blue assay (Graham 1992). We mixed 100 µL of the filtered sample extracts in 3 mL distilled water, and added 1 mL of 0.016 mol L^−1^ K_3_Fe(CN)_6_ and 1 mL of 0.02 mol L^−1^ FeCl_3_. We shook the solution for 1 min to mix it well, let it stand for 15 min, then added 5 mL of stabilizer (0.2 % Gum Arabic in 17 % H_3_PO_4_) and measured absorbance at 700 nm. We used gallic acid monohydrate (Sigma-Aldrich) as a standard. For estimation of the five flavonoids (quercetin, isoquercetin, quercitrin, kaempferitrin and kaempferol), we used high-performance liquid chromatography (HPLC) (Wang *et al.* 2012a). We injected 20 µL of filtered extracts into a Dionex ultimate 3000 series HPLC (Dionex, Sunnyvale, CA, USA) and separated compounds on a ZORBAX Eclipse C18 column (4.6 × 250 mm, 5 µm; Agilent, Santa Clara, CA, USA). We eluted the flavonoids at a constant flow of 1 mL min^−1^ with methanol–0.4 % phosphoric acid in water (56:44) and recorded absorbance at 254 nm. We estimated the concentration of each compound in a sample by peak areas of known concentrations of standards (quercetin, isoquercetin, quercitrin, kaempferol—Sigma-Aldrich; kaempferitrin—National Institutes of Food and Drug Control, Beijing, China). We calculated total flavonoids as the sum of these five flavonoid concentrations.

### Statistical analyses

We used a series of ANOVAs to examine the effects of different treatments (seven-level variable: three herbivores, two hormones, two controls), harvest time and their interaction on EFN (odds of production: binary distribution, logit link; volume), latex secretion (mass) and chemical concentrations (total phenolics, total tannins and flavonoids [total and individual]; leaf, root, root:shoot). We used adjusted means partial difference tests to examine: (i) whether a defence responded significantly to an herbivore or hormone treatment by comparing it to the appropriate control treatment, (ii) whether the strengths of plant responses differed between pairs of herbivore treatments (three pairings) or the two hormone treatments. We used custom hypothesis tests (also known as complex contrast hypothesis tests) to test whether the strengths of plant responses differed between pairs of herbivores and hormones (six pairings) as ([herbivore − no spray] − [hormone − ethanol]). We analysed the relatively large multivariate defence data set containing both indirect defences and direct defences for leaves and roots by principal component analysis (PCA) to visualize defence profiles between treatments as well as the correlations between defence types. We conducted a second PCA with above-ground and below-ground flavonoids. We conducted additional ANOVAs to examine the responses of PCA axes to induction treatments, harvest time and their interaction. We performed all data analyses with SAS (version 9.4).

## Results

**Figure 1.**
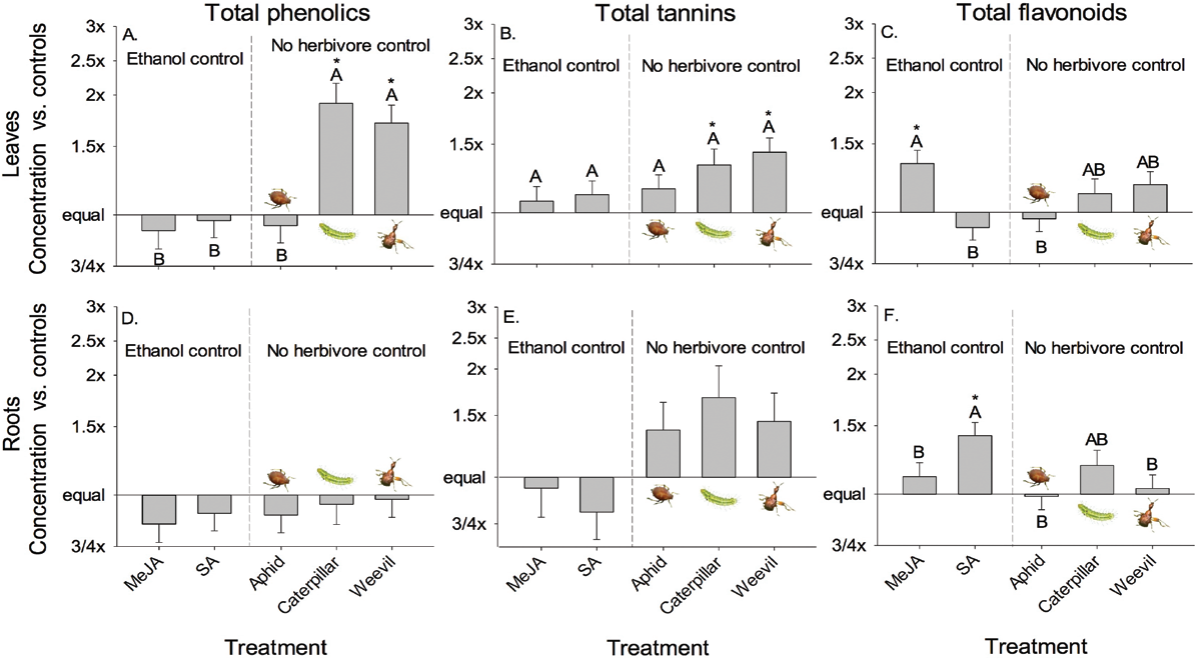
Relative concentrations of secondary metabolites with induction by exogenous hormones (MeJA; SA) and by different insects (aphid: *Toxoptera odinae*; caterpillar: *Gadirtha inexacta*; weevil: *Heterapoderopsis bicallosicollis*) compared to either an ethanol spray control or no herbivory control. (A) Leaf total phenolics (ethanol control = 6.45 mg g^−1^; no herbivore control = 5.88 mg g^−1^); (B) leaf total tannins (ethanol control = 5.48 mg g^−1^; no herbivore control = 4.19 mg g^−1^); (C) leaf total flavonoids (ethanol control = 6.86 mg g^−1^; no herbivore control = 8.95 mg g^−1^); (D) root total phenolics (ethanol control = 8.39 mg g^−1^; no herbivore control = 8.90 mg g^−1^); (E) root total tannins (ethanol control = 4.97 mg g^−1^; no herbivore control = 3.23 mg g^−1^); (F) root total flavonoids (ethanol control = 2.92 mg g^−1^; no herbivore control = 3.20 mg g^−1^). Bar height indicates relative value of a treatment mean versus the appropriate control. Associated SE values are for treatment adjusted means. Bars with the same letters were not different in strength of induction. Difference of a mean from control: *P < 0.05.

**Figure 2.**
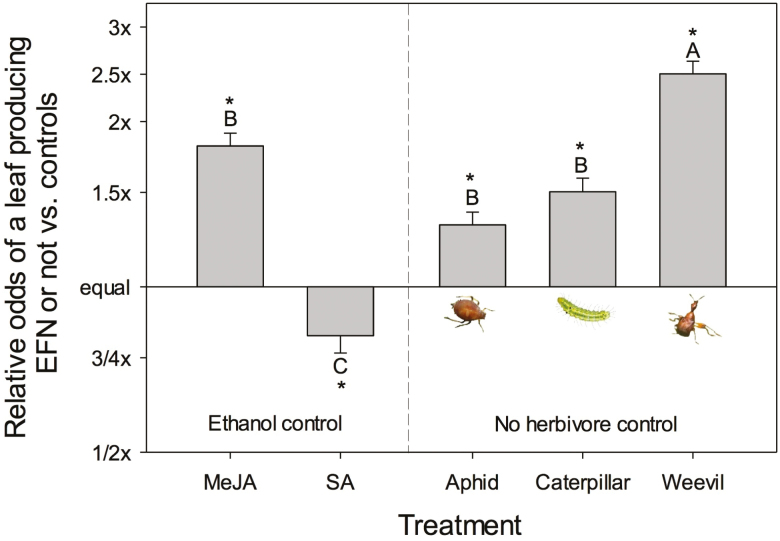
The relative odds of a leaf producing EFN or not with induction by exogenous hormones (MeJA; SA) and by different insects (aphid: *Toxoptera odinae*; caterpillar: *Gadirtha inexacta*; weevil: *Heterapoderopsis bicallosicollis*) compared to either an ethanol spray control or no herbivory control. Values are means of relative odds (log likelihood ratios) and SE versus the appropriate control (ethanol control = 0.21; no herbivore control = 0.18). Bars with the same letters were not different in strength of induction. Difference of a mean from control: *P < 0.05.

Broad categories of direct defences were induced above- and below-ground (Table 1). Chewing herbivores (caterpillars and weevils) and MeJA increased above-ground direct defences but SA increased below-ground direct defences. Leaf total phenolics and total tannins increased with chewing herbivores (Fig. 1A and B). Total phenolics and total tannins in roots did not vary with treatment (Fig. 1D and E). Chewing herbivores shifted allocation of total phenolics from roots to leaves. Leaf total flavonoids increased with MeJA (Fig. 1C) and root total flavonoids increased with SA (Fig. 1F). Salicylic acid shifted the allocation of total flavonoids to roots (Fig. 1C and F). All herbivores and MeJA increased above-ground indirect defence (EFN), with weevils inducing the strongest response, but SA decreased EFN production (Fig. 2; relative odds of a leaf producing EFN). Latex production did not differ across treatments but did vary through time **[see****Supporting Information—Fig. S2C****]**.

**Table 1.** Two-way ANOVAs showing the effects of induction treatment, harvest time and their interaction on the response of different defence types of *Triadica sebifera*; significant results are shown in bold type. Principal component analysis indicates whether a defence was included in the PCA.

Defence	Response	Treatment		Time		Treatment × time		PCA
		*F*_6, 84_	*P*	*F*_2, 84_	*P*	*F*_12, 84_	*P*	
EFN	Odds of EFN	**38.31**	**<0.0001**	**19.40**	**<0.0001**	**6.52**	**<0.0001**	X
	Volume (µL)	**5.62**	**<0.0001**	**4.38**	**0.0155**	1.44	0.1646	
Latex	Mass (mg)	1.01	0.4212	**12.73**	**<0.0001**	1.20	0.2976	X
Total phenolics	Leaf	**6.91**	**<0.0001**	**5.56**	**0.0054**	**1.92**	**0.0435**	X
	Root	0.60	0.7294	**7.12**	**0.0014**	**2.31**	**0.0133**	X
	R:S	**2.45**	**0.0311**	**8.27**	**0.0005**	1.80	0.0604	
Total tannins	Leaf	****2.73****	**0.0178**	2.93	0.0591	1.06	0.4078	X
	Root	0.81	0.5647	**8.05**	**0.0006**	1.23	0.2789	X
	R:S	0.39	0.8807	**7.18**	**0.0013**	0.70	0.7437	
Total flavonoids	Leaf	**6.20**	**<0.0001**	**3.61**	**0.0314**	1.33	0.2182	X
	Root	**2.58**	**0.0241**	**11.60**	**<0.0001**	1.02	0.4407	X
	R:S	**4.76**	**0.0003**	**7.57**	**0.0010**	1.30	0.2327	
Defence types	PCA1	**7.68**	**<0.0001**	1.05	0.3532	1.51	0.1367	
	PCA2	1.88	0.0935	**27.17**	**<0.0001**	1.39	0.1858	

**Figure 3.**
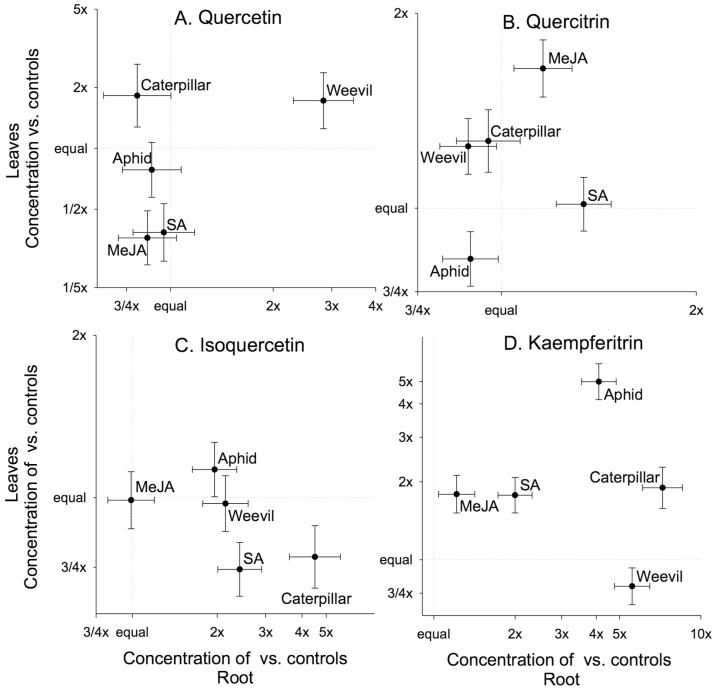
Relative concentrations of four flavonoids: (A) quercetin, (B) quercitrin, (C) isoquercetin, (D) kaempferitrin in leaves and roots with induction by exogenous hormones (MeJA; SA) and by different insects (aphid: *Toxoptera odinae*; caterpillar: *Gadirtha inexacta*; weevil: *Heterapoderopsis bicallosicollis*) compared to either an ethanol spray control or no herbivory control. Values are adjusted means ± SE versus the appropriate control. Control means listed in **Supporting Information—Table S1**.

Responses of individual flavonoids above-ground and below-ground varied with treatment (Table 2). Methyl jasmonate and SA decreased leaf quercetin and weevils increased root quercetin (Fig. 3A). Methyl jasmonate increased leaf quercitrin (Fig. 3B). Root isoquercetin increased with all but MeJA (especially for caterpillars) and the allocation of isoquercetin to roots increased with SA and chewing herbivores (Fig. 3C). Leaf kaempferitrin increased with all but weevils (especially for aphids), while root kaempferitrin increased with all but MeJA; allocation to roots increased with chewing herbivores (Fig. 3D).

**Table 2.** Two-way ANOVAs showing the effects of induction treatment, harvest time and their interaction on the response of five flavonoid types of *Triadica sebifera*; significant results are shown in bold type. Principal component analysis column indicates whether a defence was included in the PCA. Root kaempferol concentrations were below the limits of detection for many plants, so no results are shown for roots or R:S and kaempferol was not included in the PCA.

Defence	Response	Treatment		Time		Treatment × time		PCA
		*F*_6, 84_	*P*	*F*_2, 84_	*P*	*F*_12, 84_	*P*	
Quercetin	Leaf	**2.62**	**0.0224**	2.49	0.0888	**1.89**	**0.0473**	X
	Root	**5.25**	**0.0001**	0.80	0.4514	0.53	0.8864	X
	R:S	1.60	0.1568	2.70	0.0733	1.15	0.3300	
Quercitrin	Leaf	**8.27**	**<0.0001**	**7.51**	**0.0010**	1.52	0.1345	X
	Root	0.87	0.5217	**14.55**	**<0.0001**	0.71	0.7422	X
	R:S	**4.37**	**0.0007**	**18.08**	**<0.0001**	1.40	0.1825	
Isoquercetin	Leaf	**2.56**	**0.0249**	2.63	0.0779	1.46	0.1572	X
	Root	**8.49**	**<0.0001**	**11.81**	**<0.0001**	0.74	0.7057	X
	R:S	**9.48**	**<0.0001**	**13.26**	**<0.0001**	0.92	0.5276	
Kaempferitrin	Leaf	**29.11**	**<0.0001**	**8.65**	**0.0004**	1.26	0.2597	X
	Root	**20.03**	**<0.0001**	**26.24**	**<0.0001**	0.65	0.7961	X
	R:S	**13.59**	**<0.0001**	**23.61**	**<0.0001**	1.13	0.3503	
Kaempferol	Leaf	**6.27**	**<0.0001**	**9.41**	**0.0002**	1.12	0.3571	
Flavonoid types	PCA1	**7.07**	**<0.0001**	**35.67**	**<0.0001**	1.37	0.1968	
	PCA2	**15.71**	**<0.0001**	4.36	0.0157	0.91	0.5435	

**Figure 4.**
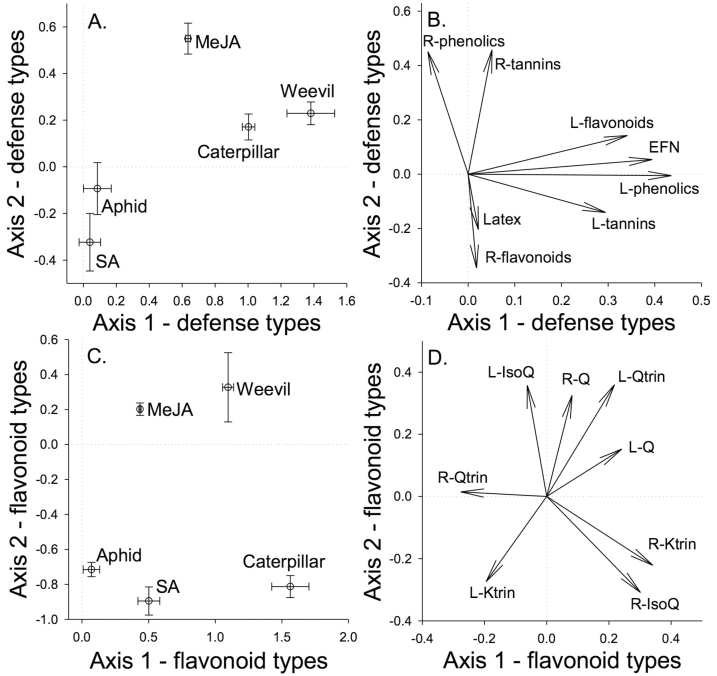
Principal component analysis scores and loadings, based on multivariate induction responses of the arithmetic differences of the factor means for a particular treatment from the appropriate control (aphid: *Toxoptera odinae*; caterpillar: *Gadirtha inexacta*; weevil: *Heterapoderopsis bicallosicollis*; MeJA: methyl jasmonate; SA: salicylic acid). (A) No herbivory control: *x* = −0.403, *y* = 0.071; ethanol control: *x* = −0.511, *y* = −0.273. (C) No herbivory control: *x* = −0.529, *y* = 0.718; ethanol control: *x* = −0.517, *y* = −0.325. The length of the arrows (B [for the PCA in A], D [for the PCA in C]) indicates the magnitude of the loading for the variable. Full defensive response and flavonoid types are listed in Tables 1 and 2.

When integrating across defence responses, plants had similar responses to chewing herbivores and MeJA, and similar responses to SA and aphids (Fig. 4A and B). The first PCA axis was associated with correlated positive responses of EFN and leaf total phenolics, total tannins, total flavonoids in response to chewing herbivores and MeJA. The second PCA axis was associated with trade-offs between root total phenolics and total tannins versus root total flavonoids, with chewing herbivores and MeJA inducing total phenolics and total tannins while SA and aphids enhanced total flavonoids.

Plants had distinct responses to each herbivore and there was evidence for systemic induction of some chemicals (quercetin), trade-offs between chemicals (quercetin versus kaempferitrin) and trade-offs for leaf versus root production (isoquercetin) (Fig. 4C and D). Overall, plant responses to aphids were most similar to the response to SA and plant response to weevils was most similar to the response to MeJA in their inductions of flavonoids.

Most defence metrics varied through time (Tables 1 and 2; **see****Supporting Information—Figs S1**–**S3**), with the exception of leaf total tannins, leaf quercetin, leaf isoquercetin and root quercetin. Several defences depended on an interaction between induction treatment and induction time, including the odds of a leaf producing EFN, leaf total phenolics, leaf quercetin and root total phenolics (Tables 1 and 2; **see****Supporting Information—Fig. S1**).

## Discussion

We found different above-ground herbivores induced diverse defensive responses including multiple chemicals in plant leaves and roots (Tables 1 and 2; Figs 1–4). The observed relationships (positive, negative or no correlation) among these different responses, such as positive leaf direct and indirect defensive responses and trade-offs between different direct responses (root total phenolics and total tannins versus root total flavonoids), were caused by different responses to induction types, indicating a high degree of specificity in plant responses to biotic damage (Fig. 4).

Damage by insects with different host ranges along with feeding modes can induce distinct plant defensive responses, with damage by chewing insects typically inducing JA-mediated responses, while feeding by piercing/sucking insects typically induces SA-mediated responses (Heil 2004; Walling 2008; Campos *et al.* 2009; Kawazu *et al.* 2012). In general, we found that above-ground direct defensive chemicals were more strongly induced by chewing herbivores than aphids (Figs 1 and 3). Antagonistic interactions are thought to be common between the JA and SA signalling pathways, which can result in trade-offs among JA- and SA-mediated defence traits (Caillaud *et al.* 2013; Haney *et al.* 2018). Overall, our results support this trend for some defensive responses but not others (Figs 3 and 4) and induction by MeJA and SA in this study did not always match induction by chewing and sucking herbivory, respectively (Fig. 3). For example, chewing damage strongly induced leaf total phenolics production, while MeJA application did not (Fig. 1). In contrast, SA application induced greater root total flavonoids production compared to controls (Fig. 1), whereas leaf total flavonoids production was induced by MeJA application. Insect herbivory did not significantly induce greater total flavonoids production in roots or leaves (Fig. 1), yet patterns for individual flavonoid inductions were highly specific to the flavonoid type and insect herbivore (Fig. 3) and rarely matched MeJA and SA treatments. Across all induction treatments, responses of individual flavonoids in above-ground and below-ground tissues were highly variable with apparent trade-offs between the flavonoids quercetin and kaempferitrin only manifesting in response to weevil herbivory (Fig. 3). We do not know how much of these responses were due to changes in production versus relocation of chemicals within a plant, but these results indicate that herbivore type can affect induced chemical trade-offs.

Extrafloral nectar secretion can be induced by JA application (Heil 2004), and our results support a JA induction and JA/SA antagonism for this defensive response (Fig. 2). Caterpillars induced similar EFN production as MeJA application, while aphids induced lower amounts than that induced by MeJA while SA application suppressed EFN production (Fig. 2). Though aphid feeding and SA application generally induce similar responses in plants (Walling 2000), the observed contrasting effects on EFN production in this study suggest they differ in specific chemical or physical stimuli associated with this indirect defensive response. In the current study, aphids and caterpillars induced similar amounts of EFN, although previously we have shown that caterpillars induced a greater EFN response (volume of nectar and sugar content) in *T. sebifera* than phloem-feeding scale insects (Carrillo *et al.* 2012). Together, these results suggest that individual components of defensive responses may be more reactive to herbivory identity than higher level measurements, e.g. the proportion of leaves producing EFN.

We found that weevil herbivory induced greater EFN production than caterpillar herbivory or any other induction treatment, despite caterpillars and weevils feeding similarly. Weevil damage also induced significantly more quercetin in roots than caterpillar damage, while caterpillars induced more isoquercetin in roots than weevils did. These contrasting patterns of induction indicate specificity in plant responses to particular herbivore cues, such as differences in damage by either herbivore species (amount or type of damage), or differences in herbivore-associated elicitors (e.g. gut microbes in oral secretions or faeces) (Bonaventure *et al.* 2011; Chung *et al.* 2013; Acevedo *et al.* 2015). Moreira *et al.* (2013) also found a greater induction of indirect defence with weevils over caterpillar damage, with damaged pine trees producing more non-volatile resin and volatile monoterpenes in response to weevil herbivory. Although we had no *a priori* expectation for stronger induction responses to weevils, it may be that the relative degree of specialization drives this difference, as the weevil, *H. bicallosicollis* is less specialized than the caterpillar *G. inexacta* (Wheeler *et al.* 2017). However, it is not possible to detect general differences in broad versus specialized feeding without replication of these herbivore types.

Systemic induction of secondary metabolites is common in plants (Wondafrash *et al.* 2013; Züst and Agrawal 2016). This represents somewhat of a paradox, as induced defences have conventionally thought to be time-sensitive responses to immediate and local attack (Haukioja 1991), and are costly to produce (Strauss *et al.* 2002), otherwise they would be produced constitutively (Karban 2011). Thus, inducing root responses to above-ground herbivore attack and vice versa could be viewed as non-optimal defence strategies. However, there may be adaptive benefits to systemic induction, including increased translocation of defensive chemicals (Erb *et al.* 2009) or priming for further attack (Frost *et al.* 2008; Martinez-Medina *et al.* 2016). It may also be that systemic induction occurs as a side effect of defences sharing common induction pathways. This may explain the apparent maladaptive response of above-ground EFN production in response to below-ground herbivory that has previously been reported (Huang *et al.* 2015; Carrillo and Siemann 2016), and suggests that some specific responses have non-specific effects on other defence traits. In this study, we found that herbivory by above-ground weevils and caterpillars significantly induced leaf total phenolics and leaf total tannins, and some specific root flavonoids (Figs 1 and 3), indicating that not all defensive responses are systemically induced in *T. sebifera*.

In this study, we also found a significant effect of time for most defensive responses, and this effect sometimes depended on herbivore identity (Tables 1 and 2; **see****Supporting Information—Figs S1**–**S3**). Plant responses might be induced quickly and this immediate induction can continue for a long period with little variation, fluctuate or stop. For instance, chemical defence compounds in corn can be rapidly induced 4–12 h after herbivory but induction ends after 24 h (Schmelz *et al.* 2003; Dafoe *et al.* 2011). Cardenolides in *Asclepias syriaca* increased nearly 3-fold in 24 h following damage, but the concentrations of cardenolides relaxed to control levels 5 days later (Malcolm and Zalucki 1996). In contrast, another study of cardenolides in *A. syriaca* showed a slight increase with caterpillars that remained almost unchanged for 3 days post caterpillar removal (Agrawal *et al.* 2014). Intriguingly, it could be that high variability in defence induction across different response types or through time is itself a defence against herbivores, potentially representing both a moving target for insects adapting to defences and creating heterogeneity in resource quality (Fernandes *et al.* 2011; Karban 2011, 2017; Wetzel *et al.* 2016; Morrell and Kessler 2017).

Considering the evolution of specificity in plant defensive responses could provide new insights for understanding the complexity of plant–herbivore interactions (Agrawal 2011). Plants are often attacked by multiple insects with varying host ranges and feeding types, and these insects can vary in their responses to similarly induced defences (Gutbrodt *et al.* 2012). Thus, plant direct and indirect defensive responses induced by the earlier attacker could affect the later feeder via secondary chemicals (Erb *et al.* 2011) or through EFN-mediated attraction of natural enemies. In this study, we found that herbivore types can drastically change the induction of direct defensive response (secondary chemicals) and indirect defensive response (EFN). Furthermore, different types of herbivores and plant hormone application (MeJA, SA) differed in their induction of defensive chemicals in roots and leaves, which influenced the relationships among these defence classes and types. These findings reflect the likely complexity of defensive trait evolution, but further evaluation of both plant and insect responses to multiple induced defences and their impact on sequence of herbivore arrivals would advance our understanding of plant defence systems, herbivore population dynamics and community components (Huang *et al.* 2017).

## Data

The data used for the analyses are also available as **Supporting Information**.

## Sources of Funding

This work was supported by National Key Research and Development Program (2017YFC1200100 to J.D.) and National Science Foundation (NSF)-China (31370404, 31770414 to J.D.).

## Contributions by the Authors

J.D. and L.X. conceived the idea. L.X. conducted the induction experiment and performed lab measurements. E.S., J.C., L.X. and J.D. conducted data analyses. All authors wrote the manuscript.

## Conflict of Interest

None declared.

## Supplementary Material

Supplementary Material 1

Supplementary Material 2

## Supporting Information

The following additional information is available in the online version of this article—

**Figure S1.** Effects of induction treatment, time since induction and their interaction on the concentrations of chemical defences in leaves and roots. Bars with the same lowercase letters indicate induction time means that were not significantly different in *post hoc* tests. MeJA = methyl jasmonate; SA = salicylic acid; aphid = *Toxoptera odinae*; caterpillar = *Gadirtha inexacta*; weevil = *Heterapoderopsis bicallosicollis*. Means ± 1 SE.

**Figure S2.** Effects of induction treatment, time since induction and their interaction on latex and extrafloral nectar (EFN) in leaves. Bars with the same lowercase letters indicate induction time means that were not significantly different in *post hoc* tests. MeJA = methyl jasmonate; SA = salicylic acid; aphid = *Toxoptera odinae*; caterpillar = *Gadirtha inexacta*; weevil = *Heterapoderopsis bicallosicollis*. Means ± 1 SE.

**Figure S3.** Effects of induction treatment, time since induction and their interaction on the concentrations of flavonoids in leaves and roots. Bars with the same lowercase letters indicate induction time means that were not significantly different in *post hoc* tests. MeJA = methyl jasmonate; SA = salicylic acid; aphid = *Toxoptera odinae*; caterpillar = *Gadirtha inexacta*; weevil = *Heterapoderopsis bicallosicollis*. Means ± 1 SE.

**Table S1.** Mean concentrations of four flavonoids (quercetin, quercitrin, isoquercetin, kaempferitrin) in leaves and roots of control treatments.
