# Supplementary material for: Herbivore-specific induction of indirect and direct defensive responses in leaves and roots
Source: AoB Plants. 2019 Feb 1;11(1):plz003. doi: 10.1093/aobpla/plz003 (PMC6378760; doi:10.1093/aobpla/plz003)
Supplement: Supplementary Material 1 [file plz003_suppl_supplementary_material_1.pdf]

Supplement

Figure S1. Effects of induction treatment, time since induction, and their interaction on the concentrations of chemical defenses in leaves and roots. Bars with the same lowercase letters indicate induction time means that were not significantly different in post-hoc tests. MeJA=Methyl jasmonate; SA= Salicylic acid; Aphid=*Toxoptera odinae*; Caterpillar= *Gadirtha inexacta*; Weevil= *Heterapoderopsis bicallosicollis*. Means ± 1 se.

Figure S2. Effects of induction treatment, time since induction, and their interaction on latex and EFN in leaves. Bars with the same lowercase letters indicate induction time means that were not significantly different in post-hoc tests. MeJA=Methyl jasmonate; SA= Salicylic acid; Aphid=*Toxoptera odinae*; Caterpillar= *Gadirtha inexacta*; Weevil= *Heterapoderopsis bicallosicollis*. Means ± 1 se.

Figure S3. Effects of induction treatment, time since induction, and their interaction on the concentrations of flavonoids in leaves and roots. Bars with the same lowercase letters indicate induction time means that were not significantly different in post-hoc tests. MeJA=Methyl jasmonate; SA= Salicylic acid; Aphid=*Toxoptera odinae*; Caterpillar= *Gadirtha inexacta*; Weevil= *Heterapoderopsis bicallosicollis*. Means ± 1 se.

Figure S1.

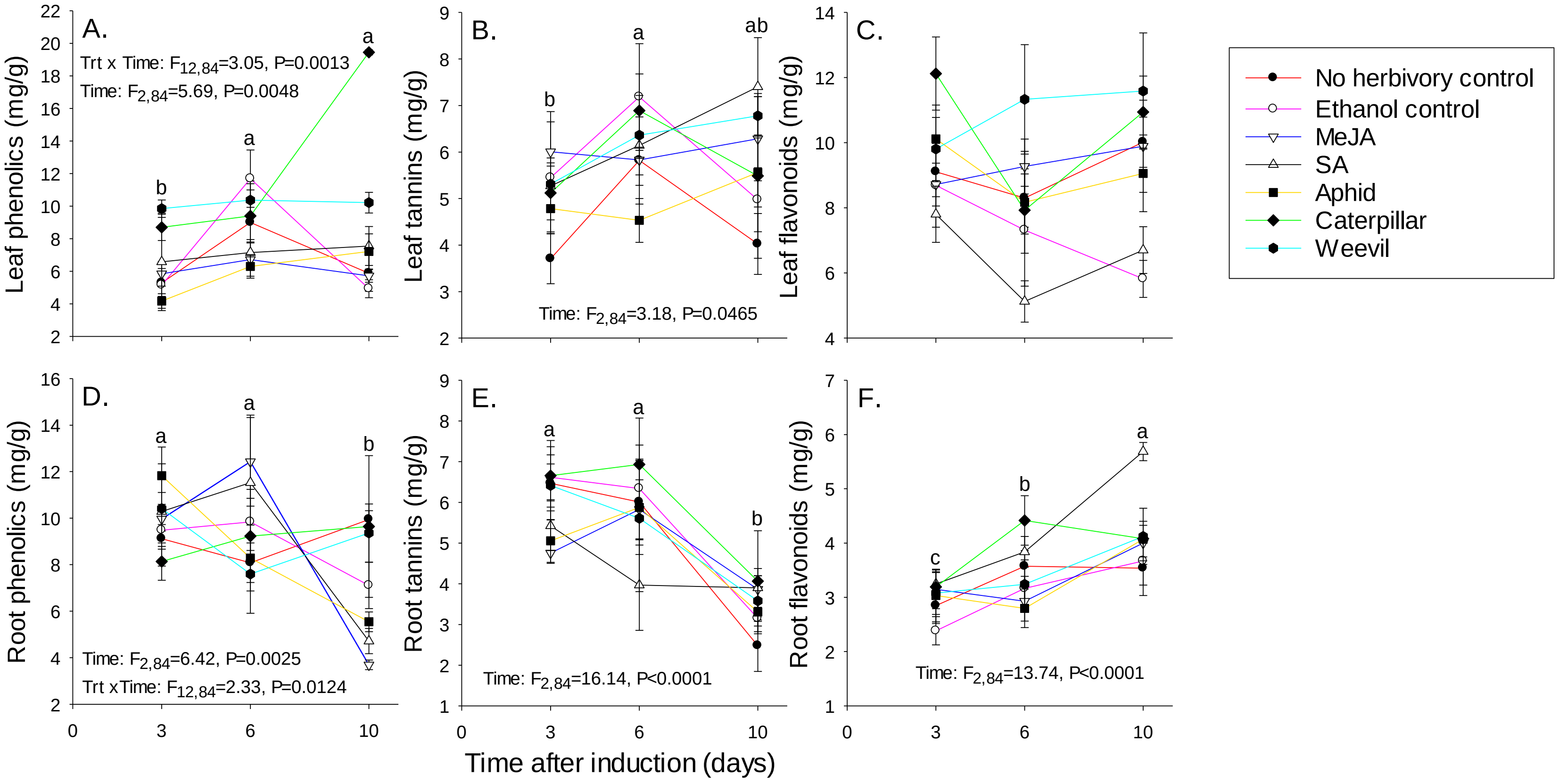

A.
Trt x Time: F12,84=3.05, P=0.0013
Time: F2,84=5.69, P=0.0048
Leaf phenolics (mg/g)
B.
Time: F2,84=3.18, P=0.0465
Leaf tannins (mg/g)
C.
Leaf flavonoids (mg/g)
No herbivory control
Ethanol control
MeJA
SA
Aphid
Caterpillar
Weevil
D.
Time: F2,84=6.42, P=0.0025
Trt xTime: F12,84=2.33, P=0.0124
Root phenolics (mg/g)
E.
Time: F2,84=16.14, P<0.0001
Root tannins (mg/g)
F.
Time: F2,84=13.74, P<0.0001
Root flavonoids (mg/g)
Time after induction (days)

Figure S2.

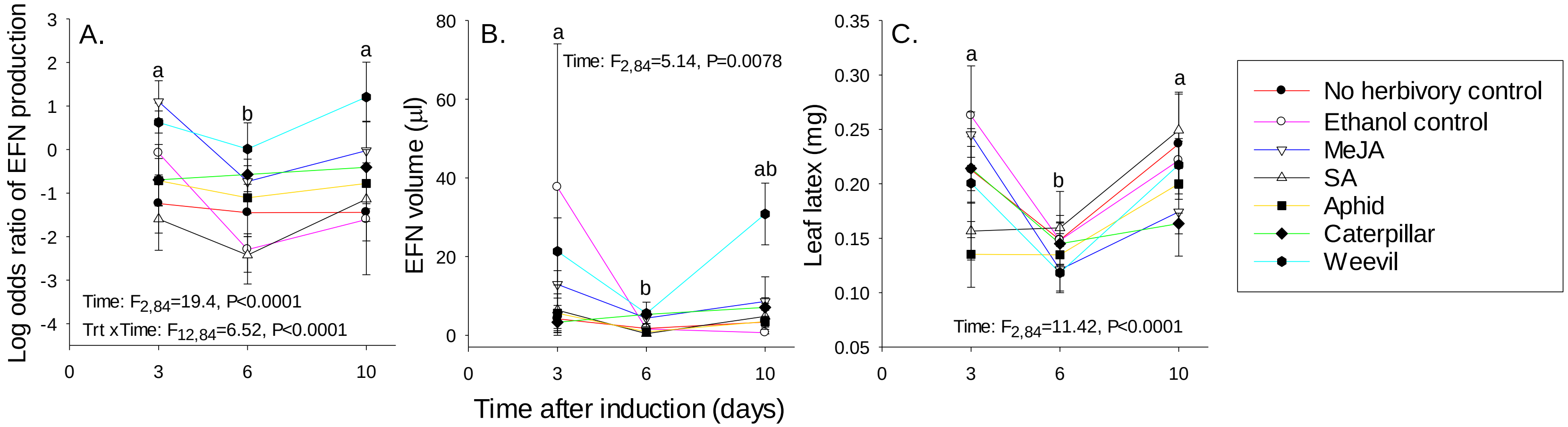

A.
B.
C.
Log odds ratio of EFN production
EFN volume (µl)
Leaf latex (mg)
Time after induction (days)
Time: F2,84=19.4, P<0.0001
Trt x Time: F12,84=6.52, P<0.0001
Time: F2,84=5.14, P=0.0078
Time: F2,84=11.42, P<0.0001
No herbivory control
Ethanol control
MeJA
SA
Aphid
Caterpillar
Weevil

Figure S3.

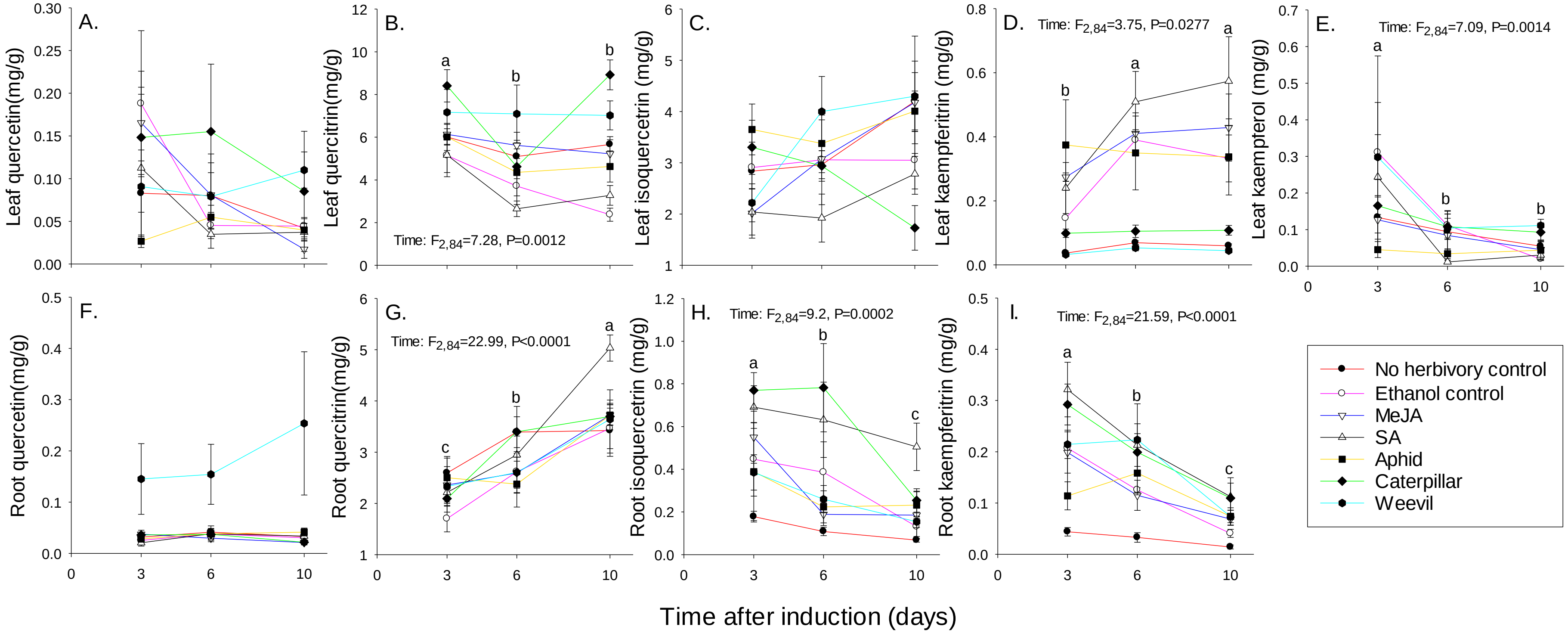
A.
Leaf quercetin(mg/g)
B.
Leaf quercitrin(mg/g)
Time: $F_{2,84}$=7.28, P=0.0012
C.
Leaf isoquercetrin (mg/g)
D.
Leaf kaempferitrin (mg/g)
Time: $F_{2,84}$=3.75, P=0.0277
E.
Leaf kaempferol (mg/g)
Time: $F_{2,84}$=7.09, P=0.0014
F.
Root quercetin(mg/g)
G.
Root quercitrin(mg/g)
Time: $F_{2,84}$=22.99, P<0.0001
H.
Root isoquercetrin (mg/g)
Time: $F_{2,84}$=9.2, P=0.0002
I.
Root kaempferitrin (mg/g)
Time: $F_{2,84}$=21.59, P<0.0001
No herbivory control
Ethanol control
MeJA
SA
Aphid
Caterpillar
Weevil
Time after induction (days)

Table S1: Mean concentrations of four flavonoids (quercetin, quercitrin, isoquercetin, kaempferitrin) in leaves and roots of control treatments.

	Leaf concentration（mg/g ）		Root concentration（mg/g ）	
	No herbivore control	Ethanol control	No herbivore control	Ethanol control
Quercetin	0.0426	0.0600	0.0332	0.0283
Quercitrin	5.3924	3.3535	3.0051	2.3303
Isoquercetin	3.1563	2.7264	0.1034	0.2139
Kaempferitrin	0.0520	0.1925	0.0239	0.0870
